# Na_3_SbSe_4−*x*_S_*x*_ as Sodium Superionic Conductors

**DOI:** 10.1038/s41598-018-27301-8

**Published:** 2018-06-14

**Authors:** Shan Xiong, Zhantao Liu, Haibo Rong, Hai Wang, Malte McDaniel, Hailong Chen

**Affiliations:** 1 The Woodruff School of Mechanical Engineering, Georgia Institute of Technology, Atlanta, GA 30332 USA; 2 College of Environment and Energy, South China University of Technology, Guangzhou, 510006 Guangdong China

## Abstract

Na based all-solid-state batteries are a promising technology for large-scale energy storage applications owing to good safety properties and low cost. High performance solid electrolyte materials with high room temperature ionic conductivity, good electrochemical stability and facile synthesis are highly desired for the commercialization of this technology. In this work, we report the synthesis and characterization of a novel fast Na-ion conductor, cubic Na_3_SbSe_4_, with an excellent ionic conductivity of 0.85 mS cm^–1^ at room temperature, and a group of S doped variants. Na_3_SbSe_4_ exhibits good compatibility with metallic Na and good stability in a wide voltage range. The application of this compound as solid electrolyte is demonstrated in all-solid-state Na-ion cells cycled at room temperature.

## Introduction

With the rapid global growth of electricity generation from renewable and clean energy sources such as solar and wind, the demand for stable, safe and low-cost energy storage solutions to flatten and level the fluctuations and intermittence in the electrical grid are becoming critical. Lithium-ion batteries (LIBs) are expensive for grid level storage due to the use of precious Li, Ni and Co metals. Sodium based electrochemical storage, such as Na-ion batteries (NIBs), are considered as a promising technology for grid storage owing to the high natural abundance and availability of sodium^1–7^. Sodium ion batteries with inorganic solid electrolyte, i.e. all-solid-state NIBs are particularly attractive in terms of safety, as no flammable organic liquid is used in the cell.

As the key component of all-solid-state NIBs, solid state Na-ion conductors with comparable room temperature conductivity as organic liquid electrolytes and good stability are very much desired. Yet in the past some super Na-ion conductors have been discovered, including *β*-alumina^8^ and NASICON type oxides^9^. However, the high grain boundary resistance in these materials commonly requires sintering at very high temperature, and high conductivity at room temperature is not easy to achieve. Recently the fast progress on chalcogenide-based alkali-metal-ion conductors have attracted a great deal of attention^10–12^. The promise has been well demonstrated first in Li-ion conductors. High ionic conductivities of 1–10 mS cm^−1^ at room temperature have been achieved in sulfides such as Li_10_GeP_2_S_12_^13^, Li_7_P_3_S_11_^14^, and Li_9.54_Si_1.74_P_1.44_S_11.7_Cl_0.3_^15^, which have enabled a number of well performing all-solid-state LIBs. Promise has also been achieved in chalcogenide sodium-ion conductors. High conductivity was computationally predicted and then experimentally achieved in Na_10_MP_2_S_12_ (M = Si, Ge, and Sn)^16,17^. A cubic Na_3_PS_4_ phase with Na+ ion conductivity greater than 0.1 mS cm^−1^ at 25 °C was reported by Tatsumisago *et al*.^18^. The conductivity of Na_3_PS_4_ was further improved by introducing Si on P sites^19^ and by Cl doping on S sites^20^. High conductivities of $\sim 1\,{{\rm{mScm}}}^{-1}$ were also reported in other compounds with similar structures, such as Na_3_PSe_4_^21,22^, Na_3_SbS_4_^23,24^ and Na_3_P_1−*x*_As_*x*_S_4_^25^. The high conductivity in these body-centered-cubic (*bcc*) based structures was proposed by Ceder *et al*.^26^ due to the existence of low barrier diffusion pathways alternatively connecting the tetrahedral and octahedral sites. It is interesting and important to explore if the diffusion barrier can be further lowered in this *bcc* structure by tuning other critical factors, such as the size of the tetrahedral and octahedral sites and the electronegativity and polarizability of the anions.

For this reason, Na_3_SbSe_4_ was chosen for our investigation. The crystal structure of Na_3_SbSe_4_ has been identified previously^27^ and it has the same space group $(I\bar{4}3m)$ as cubic Na_3_PS_4_^18^. Compared with Na_3_PS_4_, the replacement of Se at S sites and Sb at P sites may effectively enlarge the unit cell, so as to have larger size of the intermediate sites connecting the octahedral sites, which may be beneficial for the diffusion of Na ions. In addition, bigger ionic radius and higher polarizability of Se^2−^ and Sb^5+^ are also considered to be beneficial for the diffusion of alkaline ions, as demonstrated in Na_3_PSe_4_^22^ and Na_3_SbS_4_^23^. Although selenides are more expensive than sulfides and oxides, it is worth to explore their ionic conduction properties in the context of understanding the conductivity of this group of Na-ion conductors with cubic structures. In this work, cubic Na_3_SbSe_4_ was synthesized, characterized, and electrochemically tested. A group of Na_3_SbSe_4−*x*_S_*x*_ samples were also synthesized and tested. An all-solid-state sodium-ion battery using Na_3_SbSe_4_ as the electrolyte was demonstrated for the first time with reasonably well performances at room temperature. The details of the experimental results are elaborated and discussed as follows.

## Results and Discussion

### Synthesis and structural characterizations of Na_3_SbSe_4−*x*_S_*x*_

**Figure 1:**
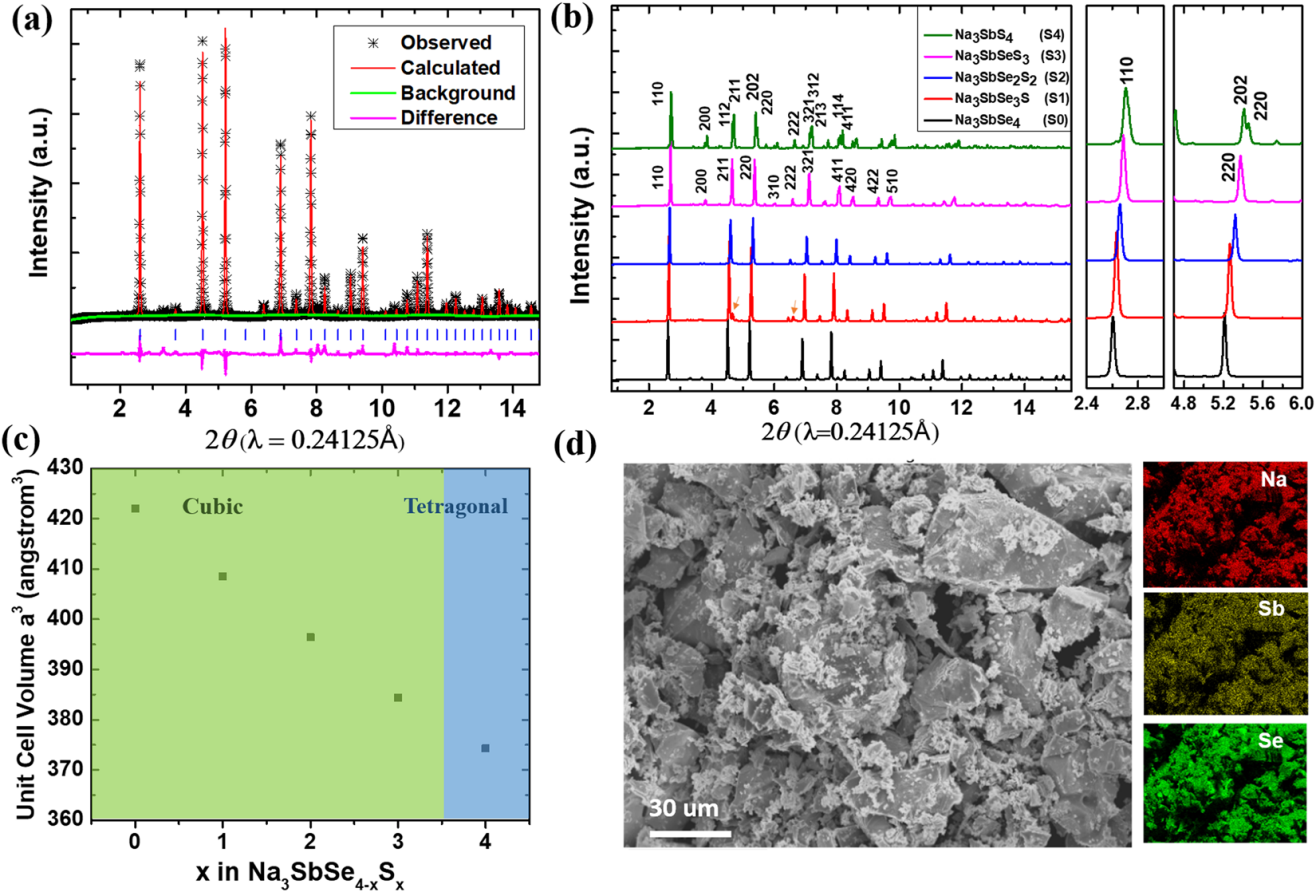
(**a**) XRD pattern of Na_3_SbSe_4_ and the Rietveld refinement. (**b**) XRD patterns of Na_3_SbSe_4−*x*_S_*x*_. (Minor impurities of NaSbSe_2_ in S1 are labeled with arrows). (**c**) Cell volume variation with respect to S concentration in Na_3_SbSe_4−*x*_S_*x*_. (**d**) SEM images of as-synthesized Na_3_SbSe_4_ and corresponding EDX mapping of Na_3_SbSe_4_.

Na_3_SbSe_4_ was successfully synthesized through calcination of the mixture of Na and Sb metals and Se powder in a sealed quartz tube at 650 °C for 18 hours. The crystal structure of Na_3_SbSe_4_ was characterized with X-ray diffraction (XRD). Figure 1a shows the synchrotron XRD pattern of as-synthesized Na_3_SbSe_4_ along with the Rietveld refinement results, which confirmed the formation of cubic Na_3_SbSe_4_ with trace amount of unknown secondary phase. A crystal structure model with space group $I\bar{4}3m$ was used for the refinement. The refined cell parameters are listed in Table 1. The unit cell has a lattice parameter of *a* = *b* = *c* = 7.501(2) Å, which is larger than that of the isostructural compounds such as Na_3_PS_4_^28^ (*a* = 6.9781 Å) and Na_3_PSe_4_^21^ (*a* = 7.3094 Å). These results are also consistent with the parameters reported by Eisenmann *et al*.^27^.

**Table 1:** Full pattern fitting refinement results for synchrotron X-ray diffraction patterns of Na_3_SbSe_4−*x*_S_*x*_ compounds.

Phase	S0 (Na_3_SbSe_4_)	S1	S2	S3	S4
Space group	$I\bar{4}3m$ (217)	$I\bar{4}3m$ (217)	$I\bar{4}3m$ (217)	$I\bar{4}3m$ (217)	$P\bar{4}{2}_{1}c$ (114)
Cell parameter	*a* = 7.501 (2) Å	*a* = 7.420 (5) Å	*a* = 7.346 (4) Å	*a* = 7.271 (7) Å	*a* = *b* = 7.163 (3) Å, *c* = 7.295 (3) Å
Cell volume (calculated)	422.04 Å^3^	408.52 Å^3^	396.42 Å^3^	384.40 Å^3^	374.30 Å^3^
R_wp_	8.83%	9.44%	7.97%	9.50%	9.38%
R_p_	6.02%	5.31%	5.75%	7.49%	6.93%
No. of variables	29	32	28	31	36

To further investigate the impact of unit cell size and anion polarizability on Na ion diffusion in this cubic structure, Na_3_SbSe_4−*x*_S_*x*_ samples with substitution of the smaller S^2−^ anion at the Se^2−^ sites were also attempted and successfully obtained using the same synthesis method. Na_3_SbSe_4−*x*_S_*x*_ samples with x = 1, 2, 3 and 4 are hereafter noted as S1, S2, S3 and S4, together with S0 noting Na_3_SbSe_4_ with no S doping. Figure 1b shows the synchrotron XRD patterns of S0 to S4. Peaks of minor impurities are observed in S1 at 2*θ* = 4.7° and 6.6°, which are from intermediate product NaSbSe_2_ and possibly due to the loss of S and Se during the heating process. With the increased S content from S0 to S4, all reflections shift toward higher two-theta angles, as more clearly defmonstrated in the zoomed area. This peak shifting indicates a gradual shrinkage of the unit cell volume, which is consistent with the substitution of Se^2−^ by the smaller S^2−^. Obvious peak splitting such as at 2*θ* = 3.82°, 4.66°, 5.44°, 6.08°, etc. is observed in S4, which indicates Na_3_SbS_4_ has a tetragonal unit cell, while others in the series are all cubic. Details of refinement results for S1, S2, S3 and S4 are also listed in Table 1. The refinement results confirmed that S1, S2 and S3 also have $I\bar{4}3m$ space group with *a* = 7.420(5) Å, 7.346(4) Å and 7.271(7) Å, respectively, while S4 was refined with a tetragonal space group $P\bar{4}{2}_{1}c$ with a slightly elongated *c*-axis (*a* = 7.163(3) Å, *c* = 7.295(3) Å and *c*/*a* = 1.018). The cell volume of the Na_3_SbSe_4−*x*_S_*x*_ phases is shown in Fig. 1c and a linear decrease as the function of S concentration is observed.

Scanning electron microscope (SEM) was used to investigate the morphology of Na_3_SbSe_4−*x*_S_*x*_ samples. Figure 1d shows the scanning electron microscope (SEM) images for the synthesized Na_3_SbSe_4_ and the elemental mapping with energy dispersive X-ray (EDX) spectroscopy. SEM images and elemental mapping for S1–S4 were shown in Figure S1 in the supplementary information. For all the compounds in this solid solution series, the particles show irregular shapes and a relatively wide distribution of particle sizes. This inhomogeneity in particle size and morphology is plausibly due to the melting-quenching process in synthesis. The distribution of Na, Sb and Se elements in the EDX mapping on the other hand confirms the uniform formation of Na_3_SbSe_4_ with no evident element or phase segregations. The elemental mappings for S1–S4 samples also show uniform distribution throughout the selected areas, indicating a series of solid-solution compounds in the designed system have been successfully obtained.

### Ionic conductivity of cubic Na_3_SbSe_4_ and Na_3_SbSe_4−*x*_S_*x*_ system

Powder of the samples was pressed into pellets and analyzed with electrochemical impedance spectroscopy (EIS). The Nyquist plots of the impedance for Na_3_SbSe_4_ at different temperatures are shown in Figure S2. The impedance spectra in the high-frequency region show a small semicircle, suggesting that the grain boundary resistance is low and the overall resistance is dominated by the bulk conductivity. This feature is similar to previously reported sulfide conductors, which typically have lower grain boundary resistance than oxides^18,21^. Based on the total resistance of Na_3_SbSe_4_ including the bulk and grain boundary resistances and the dimensions of the pellet, Na^+^ conductivity (*σ*) of the synthesized Na_3_SbSe_4_ was calculated to be 0.85 mS cm^−1^ at room temperature, which is higher than those of cubic-Na_3_PS_4_^18^, Si-doped^19^, Sn-doped^16,28^, and Se-doped^22^ cubic Na_3_PS_4_ phases. This conductivity is also comparable to those of recently reported sulfides such as Na_3_SbS_4_^23^ and Na_3_P_0.62_As_0.38_S_4_^25^. This high ionic conductivity can be explained by the large unit cell of Na_3_SbSe_4_ and the high polarizability of Se^2−^, which lowers the electrostatic binding energy and weakens the attraction between Na^+^ and the tetrahedron of [SbSe_4_^3−^]^29,30^.

**Figure 2:**
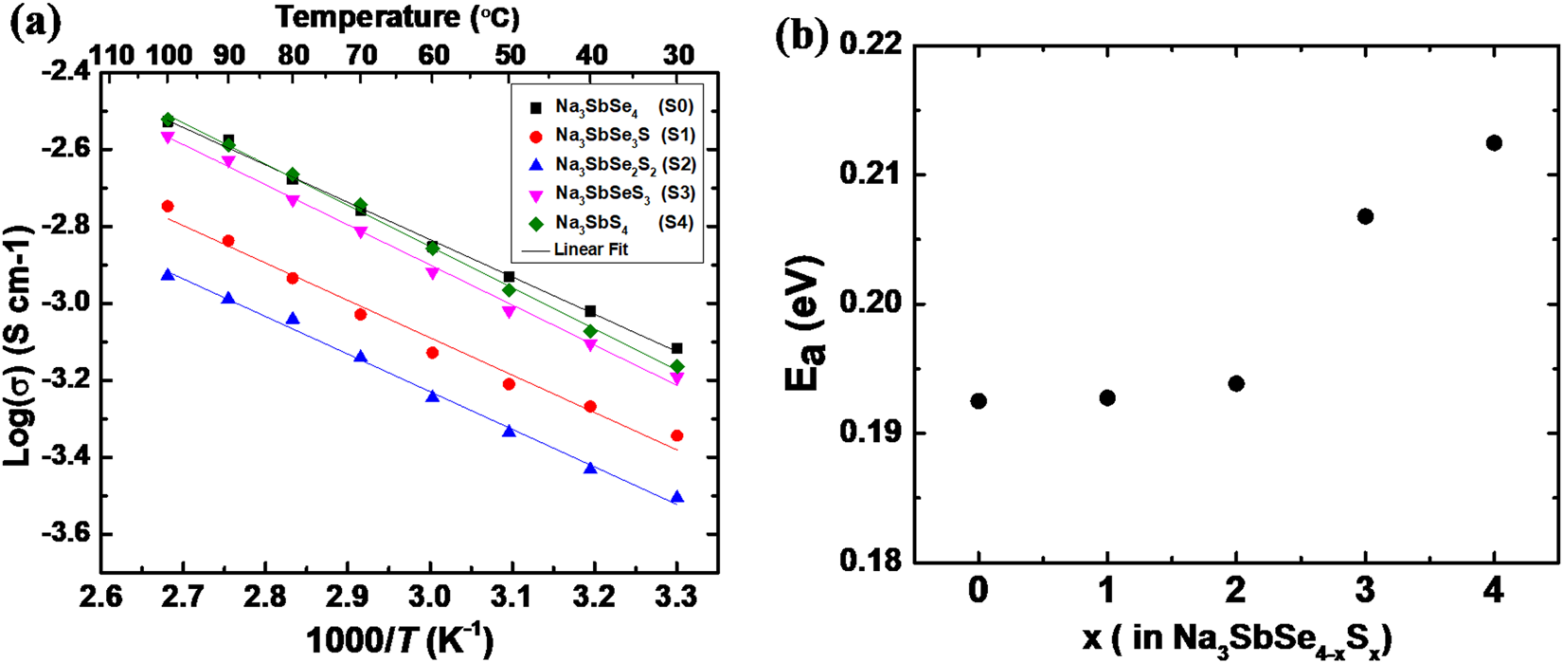
(**a**) Arrhenius conductivity plots of the Na_3_SbSe_4−*x*_S_*x*_. (**b**) the activation energies calculated from the slopes.

S0–S4 samples with different S content and therefore different unit cell sizes were all tested for ionic conductivity at variable temperatures from 30 °C to 100 °C. Arrhenius plots of the samples are shown in Fig. 2a. The log(*σ*) versus 1/T curves of all samples show good linearity, indicating no phase transition or ordering change in this temperature range. Based on the slope of the linear fitting curve, the diffusion activation energy of Na_3_SbSe_4_ is calculated to be 0.193 eV using the equation *σ* = *A*exp(−*E*_*a*_/*k*_*b*_*T*), where *A* is the pre-exponential parameter, *E*_*a*_ is the activation energy and *k*_*b*_ is the Boltzmann constant. This activation energy is lower than most of those previously reported for sulfide-based Na ionic conductors^20–25^, which ranges from 0.20–0.26 eV. With a lower activation energy, this solid-state electrolyte can maintain a relatively high conductivity at low temperature, which is significantly advantageous over organic liquid electrolytes and benefits applications in low temperature environments.

The linear fitting of conductivities of S1–S4 are also shown in Fig. 2a and the calculated activation energies are shown in Fig. 2b. With the increased S concentration in the solid solution phases, a gradual increase of activation energy can be observed for Na^+^ migration. This trend is similar to what was observed in other Se-S solid solution ionic conductors^30,31^, resulted from a softer lattice with more polarizable Se^2−^. The values of activation energies are listed in the Table S1 in supplementary information, together with the ionic conductivities of the compounds at room temperature.

Comparing the conductivities of S0–S4 samples in the variable temperature tests, it can be seen that with increased concentration of S in the solid solution, the conductivity first decreases from S0 to S2, and then increases from S2 to S4, with the two end members showing the highest conductivity. Since the sizes of the unit cell from S0 to S4 linearly increases, as shown in Fig. 1c, it is clear that the diffusivity is not solely controlled by the averaged size of the diffusion channels. The non-monotonic trend of conductivity was also reported by Yu *et al*. in the solid solution series of Na_3_P_1−*x*_As_*x*_S_4_. The highest conductivity of this series of compounds was obtained in an intermediate composition of Na_3_P_0.62_As_0.38_S_4_^25^. This effect was explained by Yu *et al*. that the dual effects of lattice expansion and a shorter Na-S bond length caused by the increased As concentration together resulted in the lowest diffusion barrier in this tetrahedral structure at the composition of As = 0.38. However, in the case of Na_3_SbSe_4−*x*_S_*x*_ series, the two end members are not in exactly the same structure. Na_3_SbSe_4_ is in cubic with an $I\bar{4}3m$ space group while Na_3_SbS_4_ is tetragonal $P\bar{4}{2}_{1}c$. The larger unit cell in Na_3_SbSe_4_ can have symmetric low barrier diffusion pathways along all three directions among Na ions sitting at 6*b* sites. On the other hand, although the unit cell size of Na_3_SbS_4_ is smaller, the tetragonal distortion plausibly provides similarly low barrier pathways along *ab* planes in the price of having higher barriers along *c*-axis^32^. Despite the lower symmetry of the tetragonal structure of S4, it shows high conductivity comparable to S0, likely due to the shorter Sb-S bonds compared with Sb-S/Se bonds in mixed anion compounds, which result in weaker Li-(S/Se) interactions and therefore facilitate faster Li diffusion. Ionic conductivity is influenced by many factors, such as structure symmetry, lattice softness, sizes of diffusion channels, bond lengths, defects, etc. At this point, it is difficult to differentiate what is the predominate factor that increases the conductivity of S4. It may be ascribed to the change of the bond length and size of the diffusion channels caused by the tetragonal distortion. As for S1–S3 samples with intermediate S concentrations and cubic structure, their smaller unit cell size than that of Na_3_SbSe_4_ and their lack of tetragonal distortion as in Na_3_SbS_4_, are likely to be the reasons for their lower conductivities than both end members.

It should also be noted that the room temperature conductivity of S4 (Na_3_SbS_4_) in this work is lower than the values reported in the recent works by Wang *et al*.^23^ and Zhang *et al*.^33^. This may be due to the different starting materials and synthesis procedures, which may introduce slight variations in amorphous phase composition, particle size, local ordering and grain boundary conditions that can have non-negligible impacts on the conductivity measurement. An additional ball-milling step prior to the impedance measurements was used in Zhang’s work to increase the conductivity of the pressed pellet. However, in our tests the ball-milled samples showed lower conductivity than as-synthesized product. This again indicates that the conductivity measurement is sensitive to many factors other than bulk phase composition and that there may still be room to further optimize and explore this series of Na_3_SbSe_4−*x*_S_*x*_ compounds for even higher conductivities. Another important issue that was not explored in current work is the local ordering of Se and S. In our model, a random mixing of Se and S at 8*c* site was assumed and it agrees very well with the refinement. However, due to the insensitivity of XRD for local structure, local ordering of Se and S cannot be excluded at this point. The local ordering may create higher (or lower) hopping barriers therefore to impact the overall conductivity. To address these issues, further investigation with other techniques, such as neutron diffraction, are undergoing in our group to reveal more information of the local ordering.

### Electrochemical properties and performance in all-solid-state batteries

**Figure 3:**
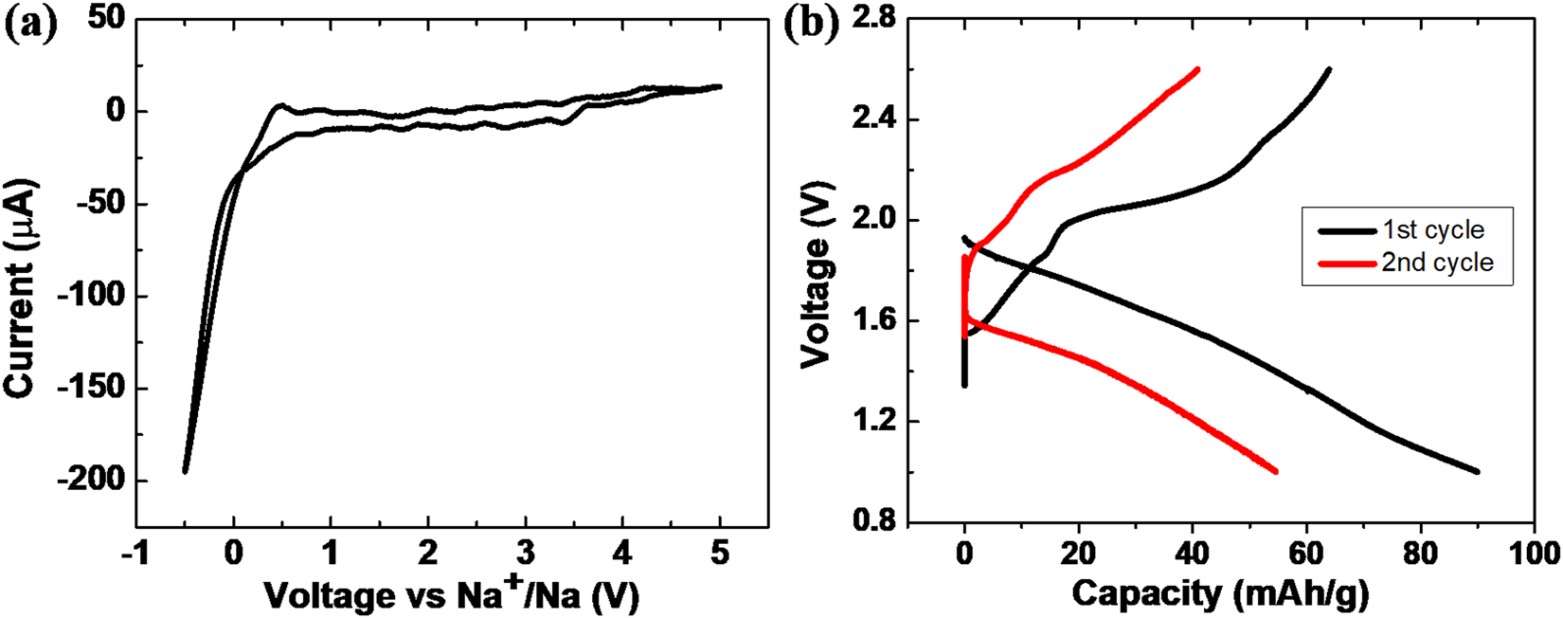
(**a**) Cyclic voltammetry curves of Na_3_SbSe_4_. (**b**) Charge–discharge curves of all-solid-state Na-Sn| Na_3_SbSe_4_| TiS_2_ cell cycled at room temperature.

The electrochemical stability of Na_3_SbSe_4_ was evaluated with cyclic voltammetry (CV) of a Na | SE | Sn cell at room temperature, with as-synthesized Na_3_SbSe_4_ as the solid electrolyte (SE), metallic Na as the reference and counter electrode, and Sn as the working electrode. In the scan from −0.5 V to 5 V at a rate of 5 mV/s, as shown in Fig. 3a, no significant current due to electrolyte decomposition is detected at high voltage. Cathodic and anodic currents are only observed near 0 V, corresponding to sodium plating and stripping. XRD patterns of Na_3_SbSe_4_ before and after CV tests are shown in Figure S3 in the supplementary information. No extra peak other than from residue Sn is observed after the CV cycles, suggesting good compatibility with metallic Na and good electrochemical stability in a wide voltage window for this material.

All-solid-state Na-ion battery Na-Sn|SE|TiS_2_ was fabricated by using synthesized Na_3_SbSe_4_ as solid electrolyte (SE), TiS_2_-SE mixed powder as cathode and Sn-Na alloy as anode. The cell was cycled at room temperature under a current density of 0.01 mA cm^−2^ (∼0.015 C based on theoretical capacity of 239 mAh/g for TiS_2_) within the voltage range from 0.9 V to 2.6 V. The charge–discharge curves were shown in Fig. 3b. The cell exhibits discharge and charge capacities of 90 mAh g^−1^ and 64 mAh g^−1^, respectively, in the first cycle. It is worth noting that the voltage profiles in solid state batteries are commonly more sloping and featureless than those in liquid electrolyte cells, due to the lower conductivity of the solid electrolyte itself and the sluggish ionic transport at the solid-solid interfaces. Similar sloping voltage curves of TiS_2_was also reported by other attempts of all solid state Na-ion batteries, such as work by Ong *et al*.^20^. The cycling curves and reasonable capacities demonstrated that Na_3_SbSe_4_ can enable all-solid-state Na-ion batteries at room temperature and the promise of the selenide-based solid electrolytes. The subsequent cycling of the cell showed relatively fast decay in capacity as the cell was far from optimized in pressure, ratio of materials and interface treatment. Although no visible degradation phases were detected by XRD, interface reactions did take place, indicated by the significantly increased impedance and a fast decay of the capacity. The interface degradation and the compatibility and stability between the electrolyte and the electrode will need further investigation. Further improvement calls for not only the optimization of the cell fabrication and test conditions, but also more fundamental understanding on the mechanical and chemical degradation of solid state batteries.

## Conclusion

In this work, a new Na ion conductor, cubic Na_3_SbSe_4_ and a group of S doped solid solution phases have been synthesized and demonstrated with high conductivities up to 0.85 mS cm^−1^ at room temperature, which is among the highest conductivities reported in chalcogenide-based Na-ion conductors. Structure characterizations reveal that the large unit cell size may be the key factor for higher conductivity than cubic Na_3_PS_4_. Na_3_SbSe_4_ also showed excellent electrochemical stability and enabled the cycling of an all-solid-state Na-ion battery at room temperature. This work will open opportunities in designing new chalcogenide ionic conductor and developing new Na-based all-solid-state batteries.

## Methods

### Synthesis of Na_3_SbSe_4−*x*_S_*x*_

Na_3_SbSe_4_ compounds were synthesized by solid-state reaction. Starting materials including Na metal (99.8%, Sigma Aldrich), Sb (99.5%, Alfa Aesar) and Se (99.99%, Sigma Aldrich) were weighed and mixed with stoichiometric ratio. Se was used with 10% excess to compensate the loss during heating. The mixture was loaded into a quartz tube in an Ar-filled glove box, and then the tube was sealed under vacuum. The tube has an inner diameter of 14 mm and a length of 8 inches. Typically, each tube contained 1 g of mixed starting materials per batch. The tube was then heated to 650 °C with a 3 °C/min ramp in a box furnace, dwelling for 18 h, and quenched on a copper plate in the ambient atmosphere. Then the tube was moved into the glove box and the product powder was obtained through hand grinding with mortar and pestle after breaking the tube. Na_3_SbSe_4−*x*_S_*x*_ (x = 1, 2, 3, 4) samples were synthesized with the same protocol by replacing stoichiometric amount of Se by S (99.5%, Alfa Aesar) in the starting materials, also with 10% excess.

### Characterizations

Crystal structures of the obtained samples were analyzed first by X-ray diffraction (XRD) with a D8 Advance X-ray Diffractometer (Bruker) equipped with a strip detector and a Molybdenum tube [*λ* K *α*_1_ = 0.7093 Å]. The powder samples were scanned in an air-tight sample container covered with a Kapton tape to avoid exposure to air and moisture. The samples were also investigated in sealed capillaries with synchrotron X-ray diffraction at beam line 17-BM at the Advanced Photon Source (APS) at the Argonne National Laboratory (ANL) and at 28-ID-2 at the National Synchrotron Light Source II at Brookhaven National Laboratory. Rietveld refinement was performed using the synchrotron XRD data with the EXPGUI suite of GSAS code^34^. Hitachi SU8230 Scanning Electron Microscope (SEM) was used to characterize the morphology and elementary distribution of the as-synthesized samples. Energy-dispersive X-ray spectroscopy (EDX) was conducted to analyze the element distribution using Oxford EDS detector with 10 kV accelerating voltage, high probe current, and 30 *μ*A emission current.

### Electrochemical Tests

AC impedance measurements were conducted using a Bio-Logic VMP3 impedance analyzer in the frequency range from 1 MHz to 0.5 Hz with a voltage amplitude of 100 mV. The pellets for the measurements were cold-pressed from as-synthesized powders at 100 MPa pressure in an acrylic tube with an inner diameter of 0.5 inch. Two stainless-steel rods were used as blocking electrodes. Temperature-dependent conductivity measurements were performed from room temperature to 100 °C with two stainless-steel rods as electrodes and a heatable pressing die with a temperature controller. Cyclic voltammetry of Na|Na_3_SbSe_4_|Sn cell was measured with Sn as the work electrode and Na metal as the counter and reference electrode.

All-solid-state Na-ion battery was fabricated using TiS_2_ (99.9%, Sigma-Aldrich) as the cathode material, Na_3_SbSe_4_ as the solid electrolyte (SE), and a Na-Sn alloy as the anode. The composite cathode consists of TiS_2_ active material and the synthesized SE powder in a weight ratio of 2:3. They were well-mixed with hand milling in an agate mortar. About 10 mg of mixed cathode powder was pressed on one side of the SE pellet (200 mg) by applying a pressure of 50 MPa in an acrylic tube with an inner diameter of 0.50 inch between two stainless-steel rods. Then a Sn foil and a Na foil were successively pressed onto the other side of the bilayer assembly by applying a pressure less than 10 MPa to form a tri-layer cell, with two stainless steel rods as the current collectors. All the fabrication processes were conducted in an Ar-filled glove box. Then the whole cell was sealed with Parafilm and Teflon tape and cycled in ambient environment. The cell was cycled galvanostatically at a current density of 0.01 mA cm^−2^ within the voltage window from 0.9 V to 2.6 V using an Arbin BT2043 battery cycler at room temperature.

## Data availability

All data analyzed during this study are included in this published article and the Supplementary Information file.

## Electronic supplementary material

Supplementary Information
