# Supplementary material for: Na3SbSe4−xSx as Sodium Superionic Conductors
Source: Sci Rep. 2018 Jun 14;8:9146. doi: 10.1038/s41598-018-27301-8 (PMC6002371; doi:10.1038/s41598-018-27301-8)
Supplement: Supplementary file 1 — Supplementary Information [file 41598_2018_27301_MOESM1_ESM.pdf]

$Na_3SbSe_{4-x}S_x$ as Sodium Superionic Conductors

Supplementary information

Shan Xiong[a], Zhantao Liu[a], Haibo Rong[a,b], Hai Wang[a], Malte McDaniel[a], Hailong Chen[a *]

[a] The Woodruff School of Mechanical Engineering, Georgia Institute of Technology, Atlanta, GA, 30332, USA.

[b] College of Environment and Energy, South China University of Technology, Guangzhou, 510006, Guangdong, China

[*] Corresponding Author. Email: hailong.chen@me.gatech.edu

Figure S1. SEM images and EDX spectroscopy elemental mappings of (a) S1, (b) S2, (c) S3, and (d) S4 in the $Na_3SbSe_{4-x}S_x$ system (corresponding to x = 1, 2, 3 and 4).

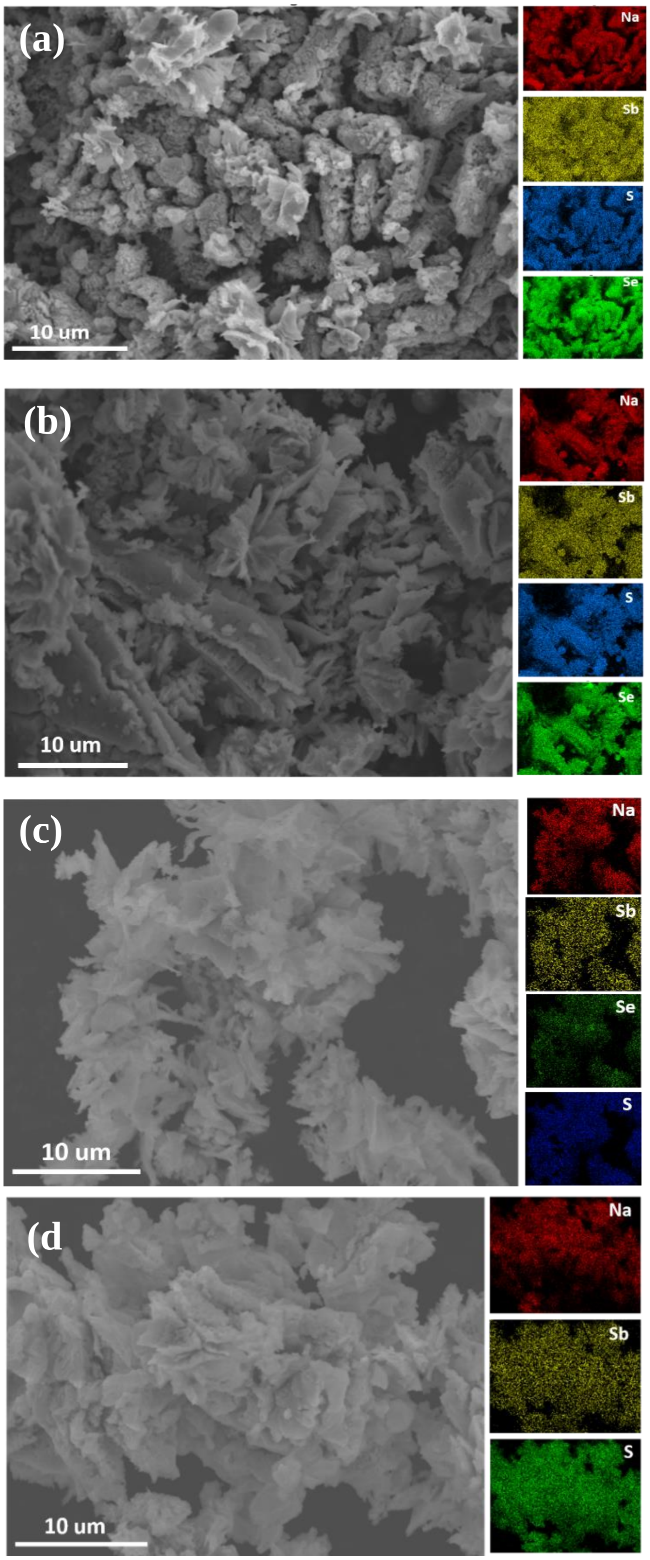

Figure S2. Nyquist impedance plot of Na_3SbSe_4 at from 30°C to 100°C.

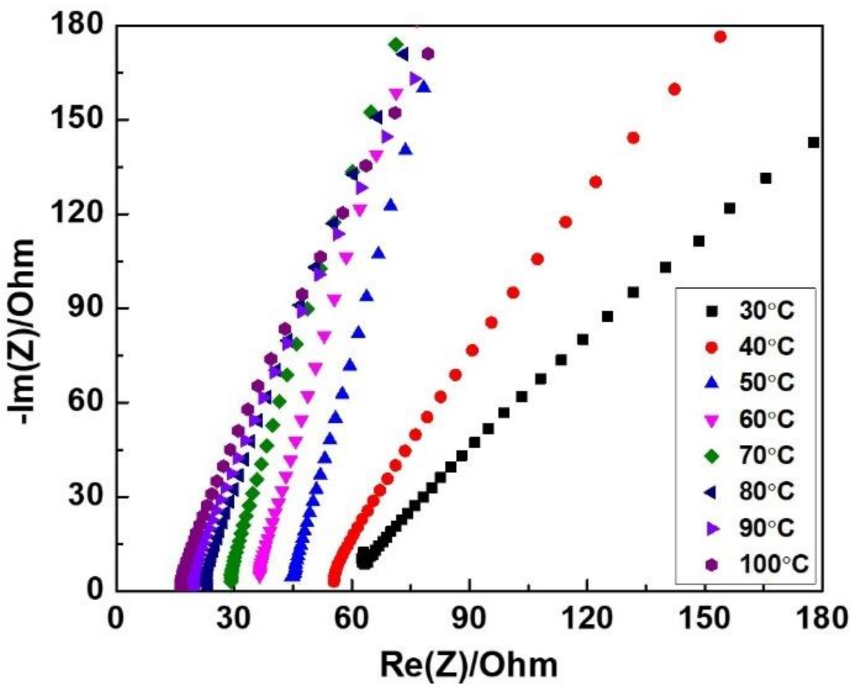

Figure S3. XRD patterns of Na_3SbSe_4 before and after CV measurements.

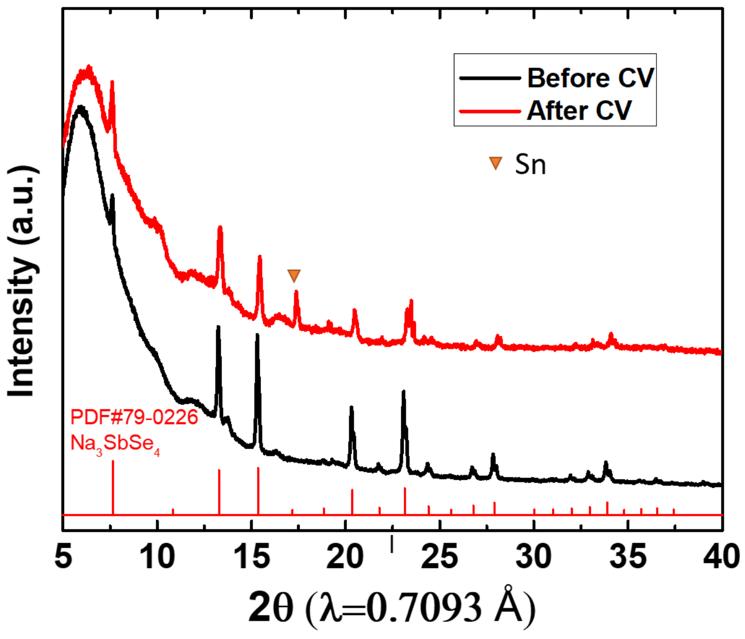

Table S1. Ionic conductivity at 30°C and activation energy of the synthesized compounds in the $Na_3SbSe_{4-x}S_x$ system.

Compound	Ionic conductivity at 30°C (mS cm^{-1})	Activation Energy (eV)
S0 (x=0)	0.85	0.193
S1 (x=1)	0.45	0.193
S2 (x=2)	0.32	0.194
S3 (x=3)	0.64	0.207
S4 (x=4)	0.69	0.213
